# Supplementary material for: Effects of glucose, ethanol and acetic acid on regulation of ADH2 gene from Lachancea fermentati
Source: PeerJ. 2016 Mar 10;4:e1751. doi: 10.7717/peerj.1751 (PMC4793307; doi:10.7717/peerj.1751)

Melt curve analysis of house-keeping gene , ACT1 in *Lachancea fermentati*


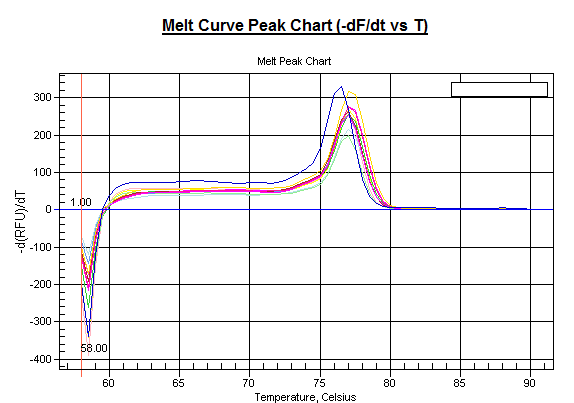


Melt curve analysis of house-keeping gene , ACT1 in *Saccharomyces cerevisiae*


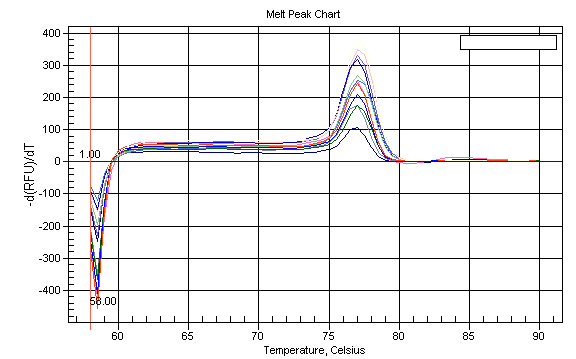


Melt curve analysis of the targeted gene , ADH2 in *Lachancea fermentati*


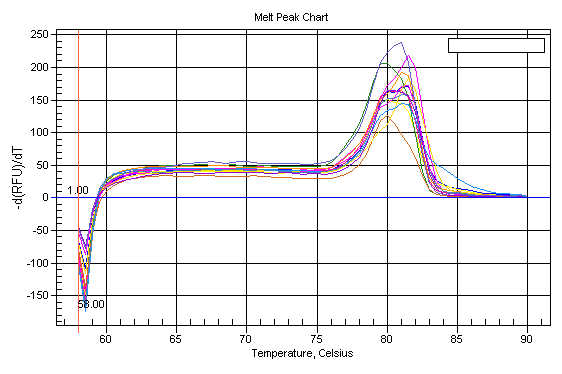


Melt curve analysis of the targeted gene , ADH2 in *Saccharomyces cerevisiae*


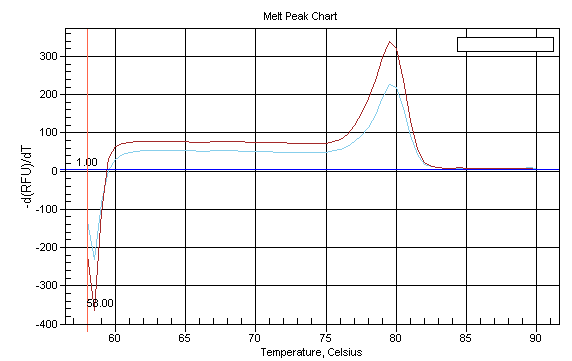

Supplement: Figure S1 [file peerj-04-1751-s002.doc]
